# New Perspectives on SNARE Function in the Yeast Minimal Endomembrane System

**DOI:** 10.3390/genes11080899

**Published:** 2020-08-06

**Authors:** James H. Grissom, Verónica A. Segarra, Richard J. Chi

**Affiliations:** 1 Department of Biological Sciences, University of North Carolina at Charlotte, Charlotte, NC 28223, USA; jgrisso9@uncc.edu; 2 Department of Biology, High Point University, High Point, NC 27268, USA; vsegarra@highpoint.edu

**Keywords:** SNARE, clathrin, yeast, endocytosis, membrane trafficking, membrane fusion, Golgi

## Abstract

*Saccharomyces cerevisiae* is one of the best model organisms for the study of endocytic membrane trafficking. While studies in mammalian cells have characterized the temporal and morphological features of the endocytic pathway, studies in budding yeast have led the way in the analysis of the endosomal trafficking machinery components and their functions. Eukaryotic endomembrane systems were thought to be highly conserved from yeast to mammals, with the fusion of plasma membrane-derived vesicles to the early or recycling endosome being a common feature. Upon endosome maturation, cargos are then sorted for reuse or degraded via the endo-lysosomal (endo-vacuolar in yeast) pathway. However, recent studies have shown that budding yeast has a minimal endomembrane system that is fundamentally different from that of mammalian cells, with plasma membrane-derived vesicles fusing directly to a trans-Golgi compartment which acts as an early endosome. Thus, the Golgi, rather than the endosome, acts as the primary acceptor of endocytic vesicles, sorting cargo to pre-vacuolar endosomes for degradation. The field must now integrate these new findings into a broader understanding of the endomembrane system across eukaryotes. This article synthesizes what we know about the machinery mediating endocytic membrane fusion with this new model for yeast endomembrane function.

## 1. Introduction

The budding yeast *Saccharomyces cerevisiae* has long been regarded as an excellent model organism for studying a wide variety of cellular processes, including endocytosis and the secretory pathway. It was the first eukaryote to have its genome fully sequenced, and many of the proteins found in yeast are conserved in mammalian cells. Additionally, yeast is particularly advantageous for mechanistic studies, as its core machinery is conserved in mammalian cells but with reduced components, making it an efficient and cost-effective model to study essential processes [1,2,3,4,5]. One of the fundamental cellular processes we have come to understand using budding yeast is membrane trafficking in the endomembrane system, which includes endocytosis and associated protein sorting pathways [6,7,8,9].

**Figure 1:**
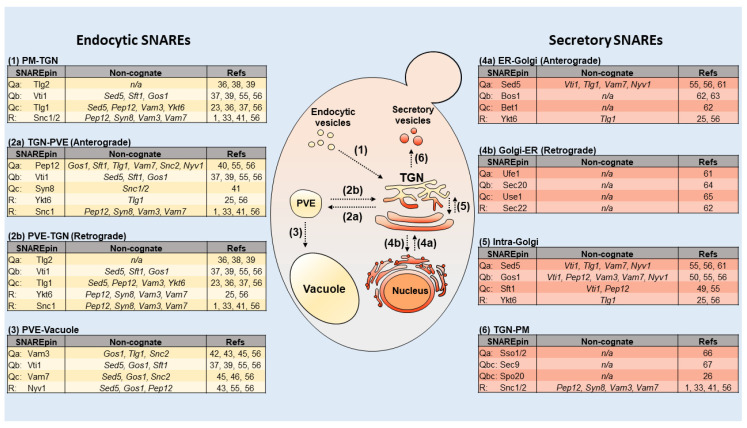
SNARE proteins in the yeast minimal endomembrane system. Center, a cartoon model depicting the yeast minimal endomembrane system. (1) In the endocytic pathway, vesicles fuse with the TGN, within 3 min post-internalization. (2) Cargo destined for degradation is trafficked to the PVE via associated sorting signals within 10 min post-internalization [16]. (2a-b) Cargo is bi-directionally trafficked between the PVE and TGN. (3) PVE cargo fuses to the vacuole for degradation within 30 min post-internalization. (4a-b) Proteins that are synthesized in the rough endoplasmic reticulum (RER) are trafficked to the Golgi. Newly synthesized proteins proceed to the TGN or are returned to the endoplasmic reticulum (ER). (5) Secretory proteins are packaged into vesicles and bud off the TGN. (6) Secretory vesicles fuse to the PM to release cargo. Moreover, v- and t-SNAREs are referred to as R-SNAREs and Q-SNAREs, respectively, due to the conserved arginine or glutamine residues found in the main interaction site of the SNAREpin core [20]. Q-SNAREs are further categorized as Qa-, Qb-, Qc- or Qbc-SNAREs, depending on the position of their SNARE motifs within the SNAREpin. Yellow indicates endocytic pathway and orange indicates the secretory pathway. Left and right tables indicate SNAREs that mediate specific fusion steps. Cognate and non-cognate interactions are also shown.

In the case of receptor- or clathrin-mediated endocytosis, cargo first binds to a plasma membrane receptor, which in turn recruits adaptor proteins and clathrin to form an inward-budding clathrin-coated pit. Once fully mature, the structure undergoes scission from the plasma membrane, forming a clathrin-coated vesicle (CCV). Next, the CCV sheds its coat before docking and fusion to the primary endocytic accepting organelle, such as the early/recycling endosome. Upon fusion, cargo is permitted to gradually dissociate from its receptor as the early endosome acidifies and matures—allowing receptor molecules to be recycled back to the plasma membrane via the *trans*-Golgi network (TGN), which acts as a recycling endosome in yeast [10]. Upon exiting the TGN, the recycled receptors return to the cell surface via the secretory pathway. As the early endosome matures, it also exchanges materials with the TGN through bidirectional fusion events [11]. Mature endosomes, also commonly referred to in yeast as the pre-vacuolar compartments or pre-vacuolar endosomes (PVE), fuse to the vacuole (which serves as a lysosome in yeast) for content degradation (Figure 1). At each step of the pathway, the docking of vesicles to the proper target endomembrane is guided by tethering factors and Rab proteins, while the fusion of the vesicle to the target membrane is mediated by membrane-bound soluble-NSF-attachment-receptor (SNARE) proteins [12,13,14,15]. Likewise, cargo within each organelle can be further sorted into other cellular compartments through pathway specific sorting motifs [16,17,18]. Multiple target-SNAREs (t-SNAREs) are located on the target membrane, and interact with vesicle-SNAREs (v-SNARE) found on the vesicular membrane. Specific t-SNARE/v-SNARE motifs are thought to bind in cognate bundles to form a hydrophobic coiled-coil structure referred to as a SNAREpin, which brings the vesicle and target membranes into contact [14,15,19]. The close proximity allows fusion between the vesicle and the target membrane.

The v-/t-SNARE interaction hypothesis was first proposed by Rothman et al. [21], and has since been at the center of our understanding of SNARE interaction and cellular distribution [15]. Different SNARE molecules are thought to reside in specific membranes, conferring specificity by mediating fusion steps only at the membranes where they are found [21]. These specific interactions are often referred to as cognate interactions, and any SNARE interactions outside of these specific pairings are referred to as non-cognate or promiscuous. For this reason, SNAREs are typically used as fluorescent markers for cell organelles. While these processes are, for the most part, well-understood, there is still uncertainty with respect to aspects of the endocytic pathway in budding yeast. For example, researchers have yet to identify a set of protein markers that distinctly label the early endosome in yeast, and there has been no evidence as to which set of SNAREs mediate endocytic vesicle fusion to the early endosome, despite the fact that the entire family of yeast SNARE proteins has been well characterized (Figure 1) [22]. One possible explanation comes from a recent study demonstrating that plasma membrane derived endocytic vesicles dock and fuse to the late Golgi, and not to recycling or early endosomes, as previously thought [23]. In this new model, yeast is proposed to have a minimal endomembrane system, in which the late Golgi functions both as an early endosome and a recycling endosome, serving as a central hub for the endo- and exocytic pathways [23]. This review article aims to reexamine studies that defined SNARE functions in yeast, and to discuss their implications for the emerging model of the yeast minimal endomembrane system and the endocytic pathway.

## 2. Overview of SNARE Function

SNAREs are a family of proteins that contain a conserved 60–70 residue SNARE motif that mediates protein-protein interactions and assembly into an α-helical bundle [15,24]. While the majority of these proteins are typically anchored to their associated membrane at their C-terminus, a small number associate to their target membranes using posttranslational modifications such as prenylation [25]. Their conserved SNARE motif is essential for vesicle fusion, as it allows for the formation of the fusion complex known as a SNAREpin [19]. In some cases, as with Sec9 and Spo20, a single SNARE is able to contribute two SNARE motifs to the complex [26]. When four SNARE motifs interact, the α-helices will entwine with one another to form a “coiled-coil” structure. The center of this coiled-coil structure is comprised of a 15-layer hydrophobic core. The center/zero layer of this core harbors an ionic rather than hydrophobic interaction between conserved glutamine and arginine residues found in the SNARE motif [20]. Furthermore, v-SNAREs and t-SNAREs are also referred to as R-SNAREs and Q-SNAREs, respectively, due to the conserved arginine or glutamine residues found in the main interaction site of the SNAREpin core [20]. Q-SNAREs are further categorized as Qa-, Qb-, and Qc-SNAREs, depending upon the position of their motifs within the SNAREpin. A SNAREpin is comprised of three t-/Q-SNARE motifs and one v-/R-SNARE motif, each providing a glutamine or an arginine to the main point of interaction in the SNAREpin (the zero ionic layer) [20,27].

**Figure 2:**
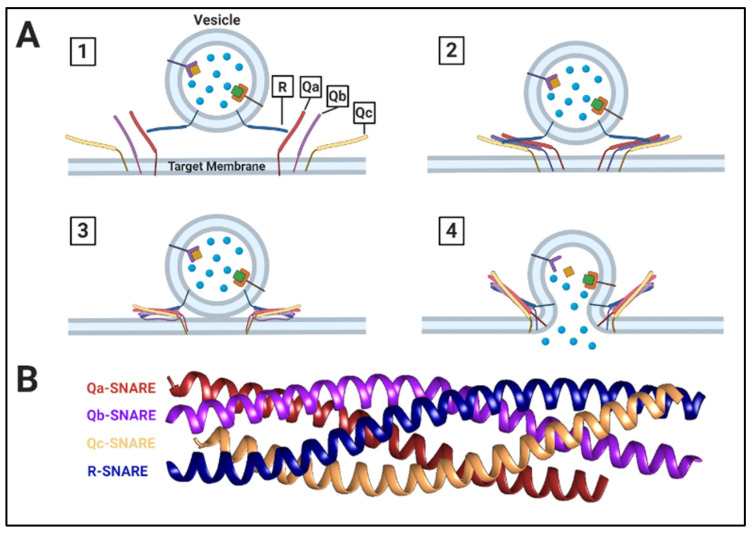
Overview of SNARE Function. (**A**) (1) Cargo-carrying vesicle with R-SNARE reaches the target membrane, which contains associated Q-SNAREs (not pictured: Rab proteins and other associated tethering factors). (2) The vesicle R-SNARE interacts with 3 target membrane Q-SNAREs to initiate SNAREpin complex formation. (3) The SNAREpin forms a “coiled-coil” quad complex, zippering the vesicle and target membrane bilayers into contact. (4) Membrane fusion occurs, releasing soluble cargo into the lumen of the target membrane. (**B**) Crystal structure of a *Saccharomyces cerevisiae* SNAREpin complex [31].

SNAREpin formation occurs once the v-SNARE engages and entwines with t-SNAREs, following the vesicle reaching its target membrane. During vesicle formation, the v-SNARE is moved from the donor to the vesicle membrane, along with other membrane proteins that will assist in mediating its fusion. Upon the vesicle reaching the target membrane, tethering factors assist in bringing these structures close enough to allow the v-SNARE and the cognate t-SNAREs to interact. The v- and t-SNAREs are unstructured prior to SNAREpin formation. As the coiled- coil structure forms, it releases energy, as the SNARE motifs are “zippered” from the N-terminus to the C-terminus acting as a catalyst, providing the majority of the force required for membrane fusion [28]. The zippering of the SNAREpin drives the membranes of the vesicle and the target into close enough proximity to enable the opening of a fusion pore, allowing cargo to be transferred from the vesicle to the target organelle (Figure 2). The speed of general SNAREpin fusion was recently calculated to be a tenth of a millisecond [29]. Following membrane fusion, the SNAREpin is disassembled by the ATPase NSF (*N*-ethylmaleimide sensitive fusion protein) and α-SNAP (soluble NSF attachment protein α) [27]. Moreover, α-SNAP first binds to the SNAREpin to create a 20S particle that is recognized and bound by the N-terminal binding domain of NSF, causing disassembly of the complex [21,30]. The now-isolated t-SNAREs are then reorganized on the acceptor membrane, while the v-SNAREs are recycled back to the appropriate donor membrane via retrograde pathways, to allow for future fusion steps to occur.

## 3. The Yeast Minimal Endomembrane System

While it has long been assumed that all eukaryotic cells contain multiple endosomal compartments [32], studies in budding yeast have failed to yield any protein markers that distinctly and uniquely label the early/recycling endosomes. Although the fluorescent lipophilic dye FM4-64 is internalized by cells at the plasma membrane and subsequently labels endocytic compartments thought to represent the yeast early endosome, it has also been shown to colocalize with the trans-Golgi marker protein Sec7 within the initial stages of endocytosis [33,34]. This indicates that the TGN may also receive bulk endocytic cargo shortly after internalization, performing at least part of the function associated with the early endosome. Recently, researchers have begun to visualize the yeast endomembrane system using both FM4-64-dependent and -independent methods. Vps8-mCherry and Sec7-mCherry enable visualization of the prevacuolar (PVE) compartment and trans-Golgi, respectively. Traditional recycling/early endosome markers, such as Tlg1, Ypt31 or Chs3 fused to GFP, have been shown to significantly colocalize with Sec7-mCherry [23]. Similarly, both FM4-64 and fluorescently labeled α-factor, a yeast mating pheromone that trafficks to the vacuole for degradation, colocalize with the Golgi marker peaking at 3 min after endocytosis, before moving to the PVE at 10 min, indicating that the Golgi serves as the early endosome in yeast [23]. Improved fluorescent markers capable of labeling key compartments continue to emerge [35], facilitating more detailed insights into the yeast endomembrane system. Most recently, a study investigating the yeast v-SNARE Snc1 has indicated that one of its interacting proteins, Rcy1, may be responsible for the delivery of endocytic plasma membrane to the TGN [1].

Collectively, these studies describe a new paradigm in which cargo-carrying vesicles, formed by the inward budding of the PM, travel and fuse directly to the Golgi, with receptors recycling back to the PM via Golgi-derived vesicles (Figure 1). Next, cargo targeted for degradation is transferred to the PVE, which acts as a late endosome, retaining cargo for degradation by the vacuole. This model addresses many of the inconsistencies from earlier models, particularly the inability of researchers to discern protein markers that uniquely label the early and recycling endosomes. This new perspective offers the simplest explanation for this issue: That early and recycling endosomes do not exist as distinct structures in *Saccharomyces cerevisiae*. In the following sections, we aim to review SNARE protein function during endocytosis, in light of the minimal endomembrane system model. SNARE proteins are known to localize and mediate vesicle fusion events with specific membranes, making them ideally suited for studies to define the yeast endomembrane system [21].

### 3.1. Candidate PM to TGN SNAREs

In this section, we discuss the candidate SNAREs that could mediate transport from the PM in the yeast minimal endomembrane system. While all yeast SNAREs have been annotated (Figure 1), interestingly, none have been identified as mediating the fusion of plasma membrane-derived vesicles to the early endosome [14,15,19,22]. However, previous studies have identified several candidates for this role, such as t-SNAREs Tlg1 and Tlg2, which colocalize with TGN markers [33,36]. Tlg1 has been shown to have a role in endosome-TGN vesicle fusion [23]. Additionally, cells ablated for Tlg1 secrete carboxypeptidase precursor 1 (p1CPY). p1CPY is first synthesized in the ER then trafficked to the cis- Golgi, where it is post-translationally modified into p2CPY and ultimately matured via proteolytic cleavage by Pep4 in the vacuole [37]. The accumulation or secretion of p1CPY typically indicates a defect in Golgi trafficking [37]. Interestingly, Tlg1 has also been shown to form a SNAREpin complex with Tlg2 and Vti1 [37]. Vti1 localizes to Golgi membranes, while Tlg2 localizes to the endosome, as well as to the Golgi [38,39]. These findings suggest that Tlg1 likely has more of a Golgi function than an endosome function. Using the fluorescent dye FM4-64, t-SNARE Tlg2 has also been shown to localize to endocytic intermediates [38]. Upon inhibition of Sec18, a protein responsible for disassembling SNARE complexes, Tlg2 forms a SNARE complex with v-SNARES Snc1/2, confirming its presence on both early endocytic structures as well as the TGN [38].

While v-SNAREs are typically recycled between target membranes and t-SNAREs typically remain only on their resident membranes, Tlg1, Tlg2, and Vti1 are unusual t-SNAREs, because they localize to multiple compartments in yeast (Figure 1). Fluorescently tagged v-SNARE Snc1 localizes to both the plasma membrane and the TGN, supporting its role in endosome-TGN vesicle fusion [33]. Interestingly, the deletion of Tlg1 or Tlg2 prevents or abrogates the plasma membrane localization of Snc1 [33]. These data indicate that Snc1 is recycled back to the plasma membrane through TGN-derived secretory vesicles. This conflicts with the traditional model in which the early endosome mediates the recycling of cargoes such as Snc1 back to the PM independently of the TGN. One explanation for this finding might be that Snc1 acts as a v-SNARE for plasma membrane-endosome vesicle fusion, undergoing a trafficking cycle that involves internalization from the plasma membrane, then transport to the early endosome and later to the TGN, before being recycled back to the plasma membrane. Given the emerging model that the TGN acts as the early endosome in yeast, an alternative explanation might be that Snc1 acts as a v-SNARE for plasma membrane-TGN vesicle fusion, undergoing a trafficking cycle that involves export from the plasma membrane, then transport to the TGN, followed by recycling back to the plasma membrane.

### 3.2. TGN and PVE SNAREs

Following the internalization of cargos, those destined for degradation continue to the pre-vacuolar endosome via TGN-derived vesicles. The SNARE complex mediating this fusion step is thought to consist of the t-SNAREs Pep12, Vti1, and Syn8, located on the PVE surface, and the v-SNARE Ykt6, found on TGN-derived vesicles. Fractionation studies have indicated that Pep12 and other cognate SNAREs coincide with late endosome markers [40]. Pep12 deletion has also been shown to inhibit the transfer of vacuolar hydrolases from the Golgi to the vacuole, but does not disrupt secretory protein transport [40]. This indicates that Pep12 plays a critical role in the fusion of TGN-derived vesicles to the late endosome/pre-vacuolar compartment. The second t-SNARE in this complex, Vti1, also localizes to pre-vacuolar membranes as well as to the Golgi [39], and subcellular sedimentation studies have revealed that Vti1 co-fractionates with Pep12. Vti1 directly interacts with Pep12, as well as with the intra-Golgi t-SNARE Sed5 [39]. Pep12 and Vti1 contribute to the formation of a functional SNAREpin with the t-SNARE Syn8. First discovered through BLAST searching, Syn8 was later revealed to be a homolog of the mammalian SNARE protein syntaxin 8 [41]. Researchers have since localized Syn8 to the late endosomes, and have found that Syn8 directly interacts with Pep12, Vti1, Snc1, and Snc2, as well as the v-SNARE Ykt6 [41].

### 3.3. PVE to Vacuole SNAREs

After reaching the late or PVE, the targeting of cargos to the vacuole is thought to be mediated by the v-SNARE Nyv1 and its cognate interactions with the t-SNAREs Vam3, Vti1, and Vam7. Nyv1 was initially found to colocalize with the vacuolar membrane protein Vma1, and additional subcellular fractionation confirmed that Vam3 is enriched on vacuolar membranes [42]. Additional vacuolar fusion experiments demonstrated that both Vam3 and Nyv1 are required for successful fusion [43]. Cells lacking Vam3 accumulate precursor CPY, indicating a role for Vam3 in mediating vesicle fusion to the vacuole [42,44]. Overexpression of either Vam7 in *vam3Δ* cells or Vam3 in v*am7Δ* cells resulted in phenotypic rescue of CPY sorting, indicating genetic interaction between the two t-SNAREs [45]. Additional co-immunoprecipitation experiments confirmed their physical interaction [45]. Finally, the addition of anti-Vam7 or anti-Vam3 antibodies directly inhibited vacuole fusion, indicating not only that they function at a similar step, but also that they mediate vesicular fusion to the vacuole [46,47].

### 3.4. Intra-Golgi SNAREs

The final group of SNAREs relevant to the yeast minimal endomembrane system is responsible for mediating intra-Golgi vesicle fusion events. This group consists of the t-SNAREs Gos1, Sed5, and Sft1, as well as the v-SNARE Ykt6. The t-SNARE Sed5 localizes to the Golgi, and Sed5 knockout results in ER to Golgi trafficking defects [48]. Moreover, *sed5* mutants also accumulate invertase, indicating that it is responsible for intra-Golgi trafficking [49]. Gos1 was first discovered through its physical interaction with Sed5. Cells ablated for Gos1 accumulate secretory protein precursors, indicating a role in intra-Golgi transport [50]. Sequence analysis has identified Gos1 as a homolog of the human t-SNARE Gos-28, and immunofluorescence experiments in HeLa cells have confirmed colocalization of the yeast and human proteins [50]. Multiple studies have reported direct interactions between Sed5 and Sft1 [25,49]. Sft1 was first discovered through its ability to suppress *sed5* temperature sensitive mutants, and *sft1* mutants also showed aggregation of invertase and CPY precursors, indicating an intra-Golgi trafficking defect [49]. Finally, the v-SNARE Ykt6 was shown to directly interact with Gos1, Sed5, and Sft1 [25]. Similar to *sed5* and *sft1* mutants, *ykt6* mutant cells are enriched for CPY precursors at non-permissive temperatures, indicating a role for Ykt6 in intra-Golgi vesicle trafficking [25].

## 4. Discussion

The recently proposed yeast minimal endomembrane system has provided a new outlook on eukaryotic endocytic processes. More importantly, this model has clarified many of the field’s past irreconcilable details, such as the lack of a universally accepted protein marker for early/recycling endosomes and lack of clarity of the initial steps of fusion during endocytosis. While many of field’s past interpretations were primarily based on mammalian endo-lysosomal systems, the field must now continue to examine how this new perspective fits into all that is known about endocytic processes. In this new perspective, the yeast endomembrane system is thought to be more similar to plants with the TGN, acting as the primary acceptor membrane during endocytosis. Moreover, it is likely that, while earlier ancestral endomembrane systems were even more simplified, animals evolved a highly complex system to accommodate the influx of cellular cargos and evolved multiple pathways for material exchange [51]. This view is supported by the fact that many human and yeast SNAREs can participate in multiple fusion steps [52,53,54]. However, the exact mechanism through which SNARE proteins can participate in multiple fusion steps or pathways is not known. Below, we describe three potential mechanisms that could explain this SNARE multiplicity of function.

First, many SNAREs are promiscuous binders, and are able to associate in non-cognate combinations [55]. Interestingly, both cognate and non-cognate SNARE complexes are fully functional, and are efficient in lipid fusion experiments [55]. Interestingly, ER SNAREs have been shown to be relatively selective, while endosomal and vacuolar SNAREs are thought to be more promiscuous [56] (Figure 1). For example, the t-SNARE Vti1 is able to form functional SNAREpins for at least three separate fusion steps in the endocytic pathway, and has been localized to multiple subcellular structures in yeast and human cells [37,39,55,56,57,58,59,60]. Golgi t-SNARE Sed5 has been shown to form non-cognate SNAREpins with t-SNAREs Vti1, Tlg1, and v-SNARE Snc2, and therefore may mediate initial plasma-membraned derived vesicles for fusion [56]. Sed5 has also been shown to form a non-cognate SNAREpin with the vacuolar SNAREs Vam7, Vti1, and Nyv1 [56]. Gos1, another intra-Golgi t-SNARE, has been shown to form a complete SNAREpin with vacuolar SNAREs, and pairwise interactions with Vti1, as well as the endosomal t-SNARE Pep12 [55,56]. The final intra-Golgi t-SNARE, Sft1, shows similar promiscuous interactions with Vti1 and Pep12 [55]. The v-SNARE Ykt6, responsible for intra-Golgi and TGN-PVE trafficking, can interact with the TGN t-SNARE Tlg1 [56]. Endosomal SNAREs show a similar degree of promiscuity. In addition to interactions with intra-Golgi SNAREs, the endosomal t-SNARE Pep12 interacts with vacuolar SNAREs Vam7 and Nyv1, as well as the plasma membrane v-SNARE Snc2 [55,56]. Additionally, Pep12 has been shown to form a non-cognate SNAREpin with Vti1, Tlg1, and Snc2 [56]. Syn8, another endosomal t-SNARE, only shows promiscuous interactions with the plasma membrane v-SNAREs Snc1/2 [41]. Finally, the vacuolar t-SNAREs Vam3, Vti1, and Vam7 have also been shown to form non-cognate interactions with v-SNARE Snc2 [56]. While, in many cases, non-cognate binding may simply be due to similar cognate pair sequences and structures, the precise mechanisms are not known. We believe the high degree of SNARE promiscuity at the TGN strongly supports the minimal endomembrane model. That is, if the TGN sorts both endocytic and secretory cargo, we would expect to see overlap between SNAREs residing on those structures. In contrast, the ER to Golgi and TGN to PM secretory pathways are more clearly defined and exhibit less SNARE promiscuity [26,61,62,63,64,65,66,67] (Figure 1).

Second, regulatory SNARE-interacting proteins such as the Rab family could also be regulating vesicular and target membranes. The Rab family of GTPases are critical for SNARE complex formation, play essential roles in docking vesicles to their target membrane. They are also found in multiple pathways in the cell [68,69,70,71]. Therefore, they are excellent candidates to coordinate SNARE function in multiple fusion events. However, Rab proteins have been found to be nonselective with their respective SNAREs interactions [72], and recent reports indicate that Rabs proteins appear to be compartment-specific rather than transport-step-specific [73]. If this is the case, the Rab proteins could be better targets for organelle markers than SNAREs. Interestingly, there are only eleven Rab proteins in budding yeast, while in metazoans, there are at least sixty [68,69]. Therefore, it is also interesting to speculate about the evolutionary context for the high amounts of Rab gene duplication in mammalian cells, and their possible requirement in regulating endomembrane trafficking.

Third, regulatory post-translational modifications (PTMs) on SNAREs could facilitate complex formation between specific molecules. In fact, in vitro, many of the human SNARE proteins have been shown to contain PTMs [74,75,76]. These PTMs could in turn regulate SNARE complex formation. For example, the phosphorylation of SNAP-25, a human t-SNARE found on the plasma membrane, directly diminished its ability to interact with syntaxin to form a competent SNAREpin [77]. In yeast, the phosphorylation of the t-SNAREs Sso1 and Sec9 directly affects the binding ability of SNARE inhibitory factors that prevent the formation of SNAREpin complexes [78]. Therefore, it is highly likely that other yeast SNAREs also harbor PTMs that regulate function, however, more research on this topic is needed.
